# Supplementary material for: Actomyosin meshwork mechanosensing enables tissue shape to orient cell force
Source: Nat Commun. 2017 May 15;8:15014. doi: 10.1038/ncomms15014 (PMC5440693; doi:10.1038/ncomms15014)
Supplement: Supplementary Information — Supplementary Figures, Supplementary Methods, Supplementary Tables and Supplementary References [file ncomms15014-s1.pdf]

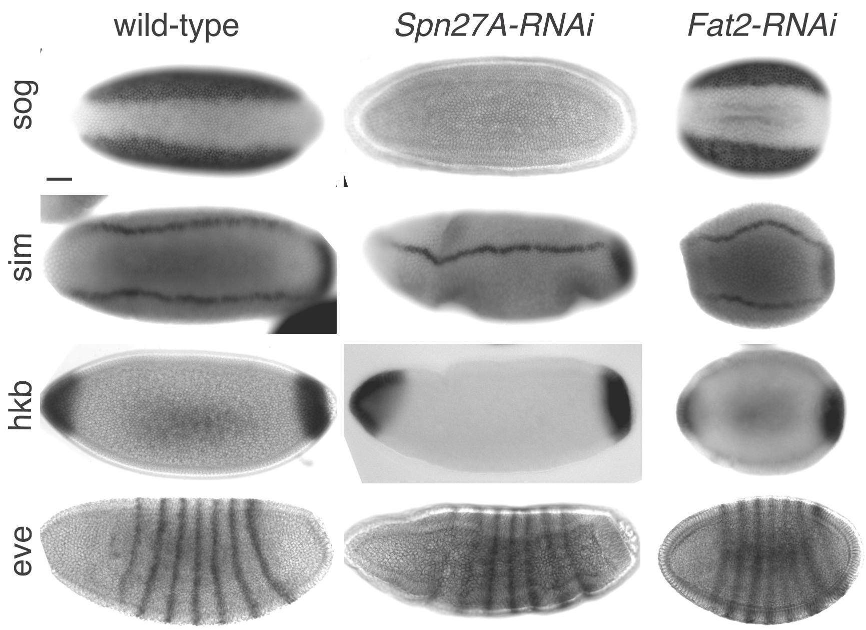

Supplementary Figure 1: ***Spn27A-RNAi* embryos are partially ventralized, whereas patterning is not affected in *Fat2-RNAi* embryos.**

RNA in situ hybridization of wild-type, *Spn27A-RNAi* and *Fat2-RNAi* embryos using probes for early patterning genes. sog expression marked cells adopting a lateral ectodermal fate (sog is repressed in the mesoderm). sim expression marked mesectoderm cells, one row of cells between the mesoderm and the ectoderm. hkb expression marked cells adopting the anterior and posterior terminal fate. eve expression participates in patterning the embryo in the a-p axis, it is expressed in seven stripes. Ventral views of the embryos are shown, except for *Spn27A-RNAi* stained with sim and eve, where lateral views are shown. Note that *Spn27A-RNA*i embryos are partially ventralized, with an expansion of the ventral fate at the expense of dorsal fates whereas lateral fates are shifted dorsally. The a-p patterning however is not affected. *Fat2-RNAi* does not affect patterning. Scale bar, 40 μm.

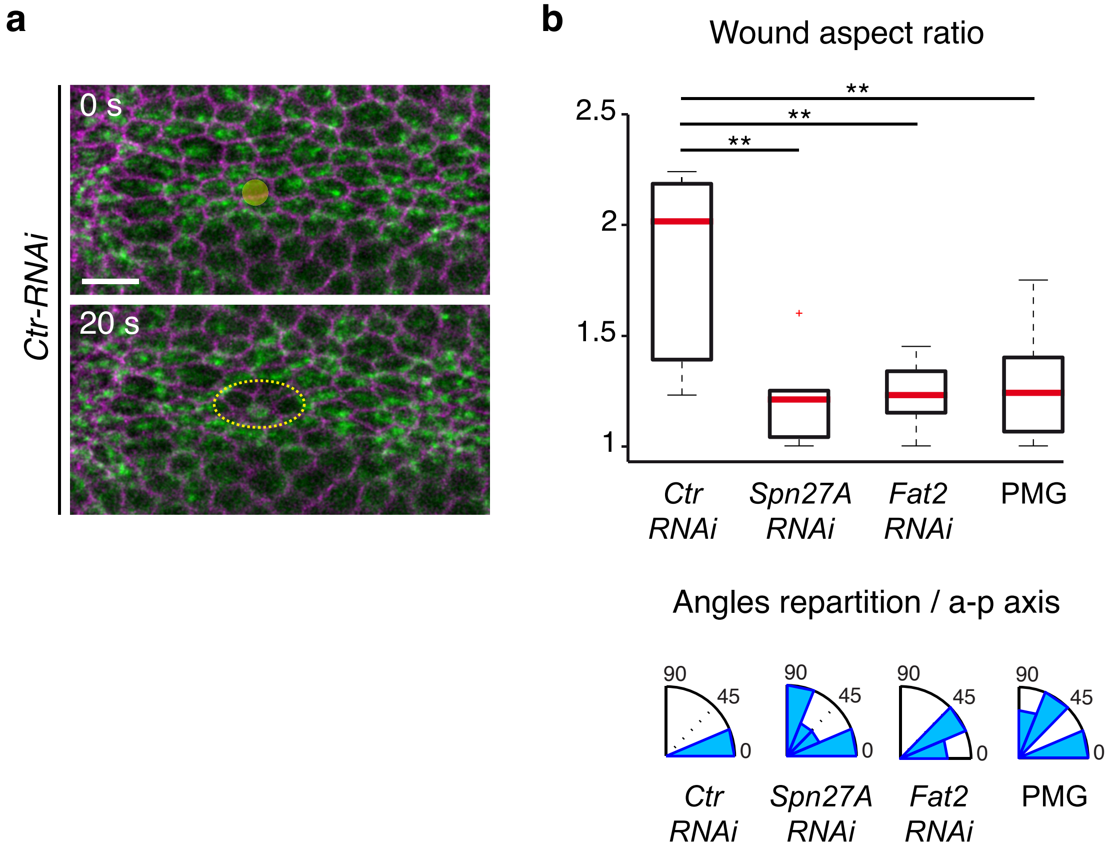

Supplementary Figure 2: **Epithelial tension is isotropic in *Spn27A-RNAi* and *Fat2-RNAi* embryos during VF formation and during PMG formation**

a. Images from live embryos before (0s) and 20s after induction of an isotropic wound (yellow discs) during VF formation in *Ctr-RNAi* embryo. Scale bar, 10 μm.

b. Box-and-whisker plot of wound aspect ratio 20 seconds after ablation (top) performed in *Ctr-RNAi* (n=8), *Spn27A-RNAi* (n=6), *Fat2-RNAi* (n=5) embryos, and the PMG (n=8). **, $p<0.01$, *t*-test. Repartition of the angles made between the fitted ellipse major axis and the embryo a-p axis (bottom).

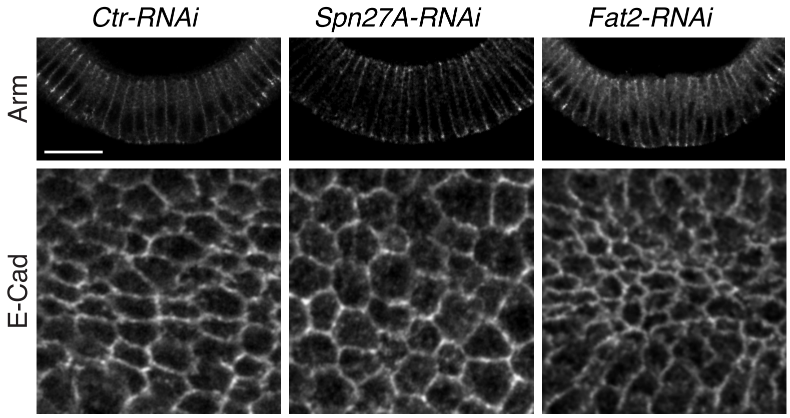

Supplementary Figure 3: **Adherens junctions are correctly assembled and positioned in *Spn27A-RNAi* and *Fat2-RNAi*.**

Top panels, cross-sections of fixed embryos stained with β-catenin (Armadillo, Arm). Arm localized in basal and subapical junctions in ectodermal cells, subapical junctions are shifted apically in mesodermal cells at the onset of gastrulation. Scale bar, 30 µm. Lower panels, en face views of VF cells stained with E-Cadherin (E-Cad) showing the belt-like organization of apical AJs. Scale bar, 10 µm.

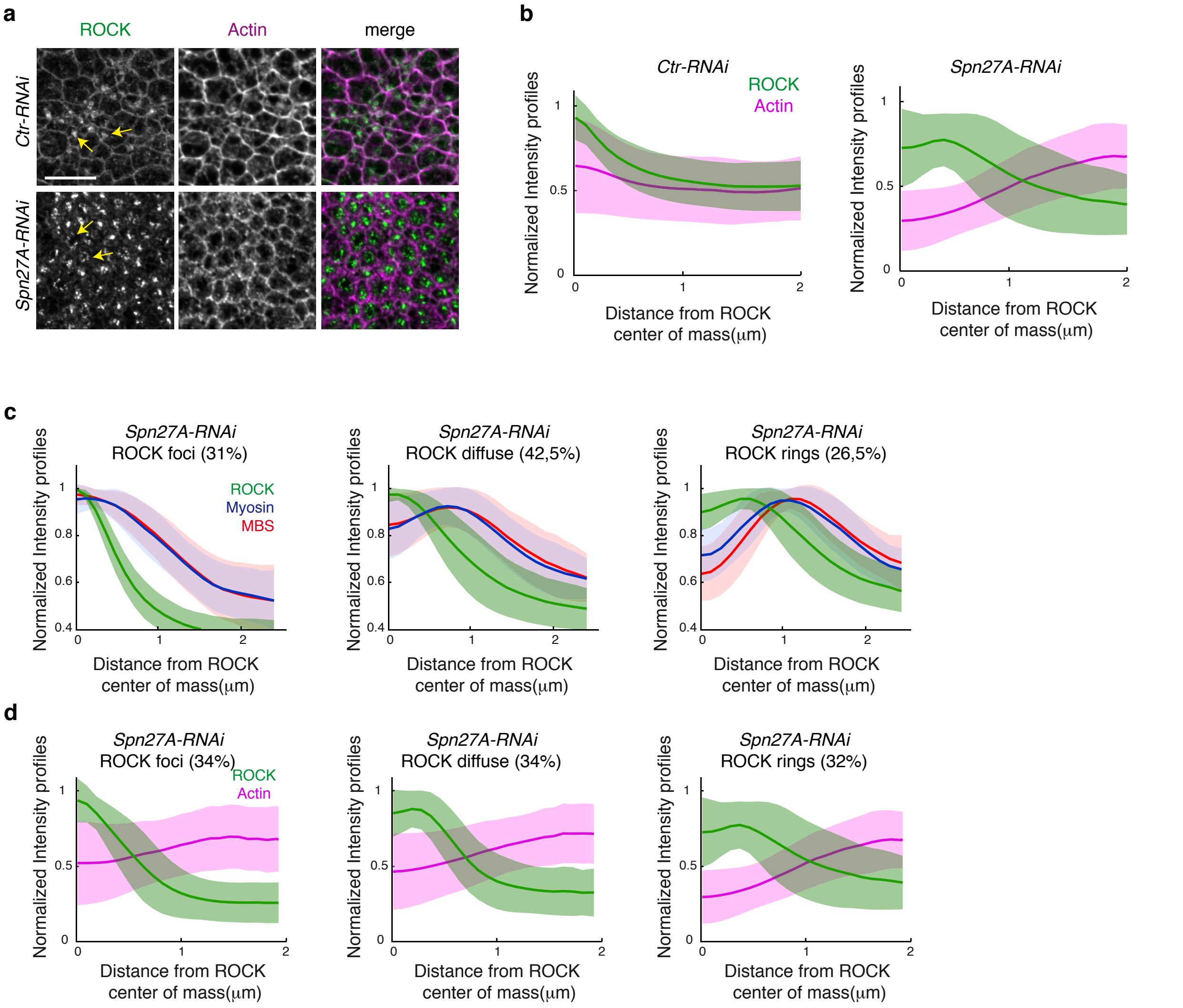

Supplementary Figure 4: **Identification of ring pattern and organization of actin and myosin in ring in *Spn27A-RNAi* cells**

a. Images of VF cells in *Ctr-RNAi* and *Spn27A-RNAi* fixed embryos expressing ROCK::GFP (green) and stained with phalloidin (F-actin, magenta). Yellows arrows point to ROCK foci in control cells and ROCK rings in *Spn27A-RNAi* cells.

b. Mean normalized intensity profile of ROCK and F-actin in *Ctr-RNAi* cells (n = 154 cells one embryo) and *Spn27A-RNAi* (n = 83 cells, one embryo) showing F-actin depletion from center of the ROCK rings, similar to myosin. Scale bars, 10 μm.

c. Mean normalized intensity profile of ROCK, MBS and myosin in *Spn27A-RNAi* cells (n = 101

cells one embryo). 27 cells fall into the ROCK ring category (right, also shown in Fig. 3d, 26.5%), 42 cells classified as diffuse ROCK (42.5%), and 31 classified as condensed ROCK foci (31%). Diffuse ROCK and rings (representing 69% of cells in *Spn27A-RNAi*) were always associated with clear myosin and MBS rings positioned outside the ring of ROCK.

d. Mean normalized intensity profile of ROCK and F-actin in *Spn27A-RNAi* cells (n = 259 cells one embryo). 83 cells fall into the ROCK ring category (right, 32%, also show in panel b.), 88 into diffuse ROCK (34%) and 88 into ROCK condensed foci (34%).

Shaded area indicates SD.

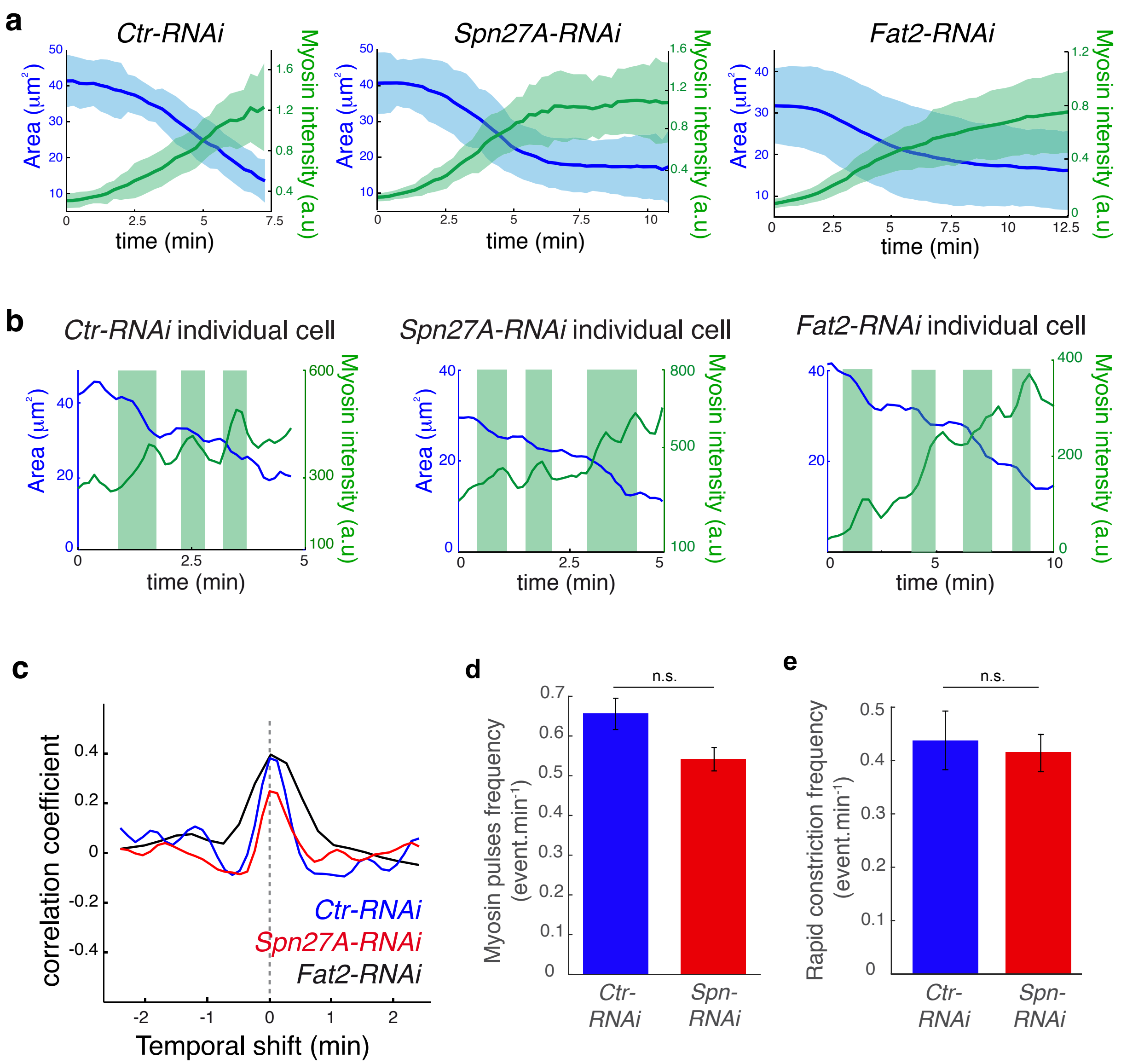

Supplementary Figure 5: **Myosin intensity increases normally and displays pulsatile behavior in *Spn27A-RNAi* and *Fat2-RNAi* embryos.**

a. Mean apical area (blue) and mean myosin intensity (green) over time for one *Ctr-RNAi* embryo (n = 71 cells), one *Spn27A-RNAi* embryo (n = 69 cells) and one *Fat2-RNAi* embryo (n = 170 cells). Shaded area indicates SD.

b. Apical area (blue) and myosin intensity (green) over time in an individual representative cell from *Ctr-RNAi*, *Spn27A-RNAi* and *Fat2-RNAi* embryos; contraction pulses are highlighted in green boxes.

c. Plot of averaged cross-correlation between constriction rate and myosin intensity rate of change (n = 59 *Ctr-RNAi* cells, n = 51 *Spn27A-RNAi* cells, n = 171 *Fat2-RNAi* cells) against temporal shift. Correlation coefficients were calculated for various temporal offsets by temporally shifting the data sets relative to each other. Note the cross-correlation peak around 0 offset in *Ctr-RNAi* (blue), *Spn27A-RNAi* (red) and *Fat2-RNAi* (black) indicating that myosin pulses correlate with a reduction in apical area.

d. Quantication of frequency of instances where there is a rapid increase in myosin intensity defined as a pulse

e. Quantification of frequency of instances of rapid apical area reduction.

Ctr-RNAi and *Spn27A-RNAi* have similar pulse frequency

n = 59 *Ctr-RNAi* cells; n = 53 *Spn27A-RNAi* cells. Error bars are SEM. n.s., not significant ($p>0.01$).

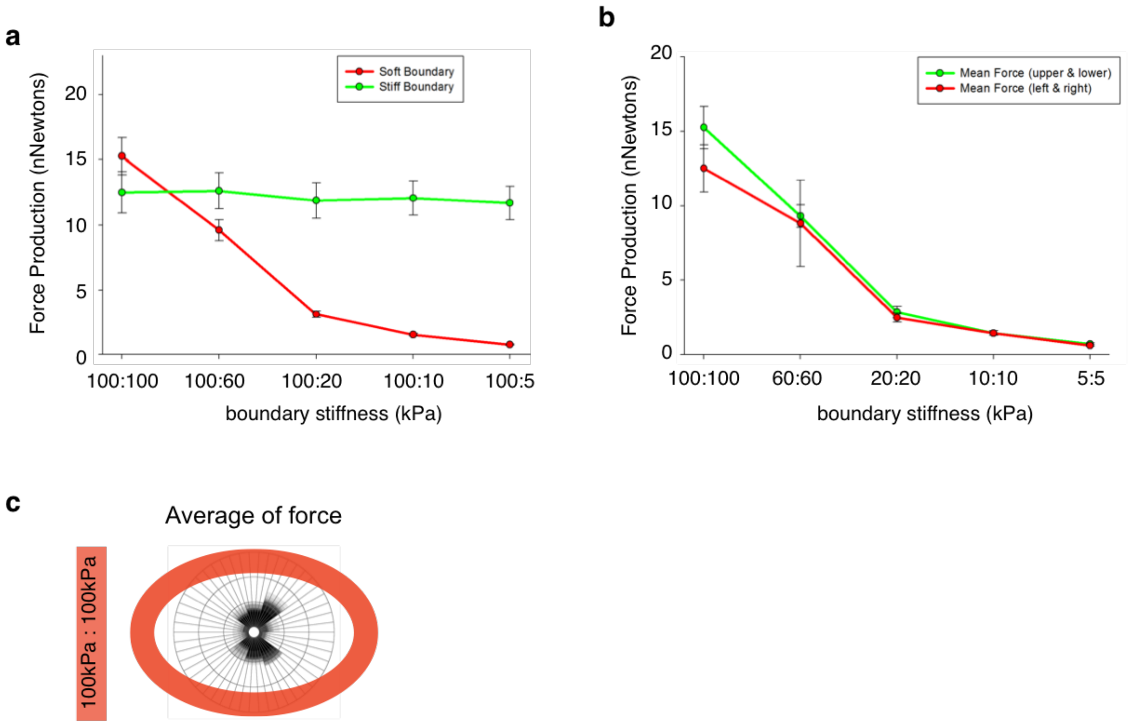

Supplementary Figure 6: **Force production by a 2D actomyosin meshwork depends on boundary stiffness but is not oriented by cell shape**

a. force production along soft boundary (green) and along stiff boundary (red) averaged for n≥10 simulations depending on the asymmetry in boundary stiffness. Difference at the 100:100 ratio represents symmetry breaking and the force is not oriented along a specific axis. As anisotropy in stiffness increases the ratio increase revealing the emergence of force directionality (always along the stiff axis). Error bars represent SD.

b. Force production depends on the stiffness of the boundary spring. Force production along the soft (green) and stiff (red) boundaries averaged for n≥10 simulations depending on the decreasing boundary stiffness. Error bars represent SD.

c. To test confinement on force production, the simulations were embeded within an elipse with an aspect ratio=2 (ellipse major radius=2 μm; ellipse minor radius=1 μm). The rose diagram illustrates the average force generated on boundary springs for 10 simulations, circles divisions on the rose diagrams represent 10 nN.

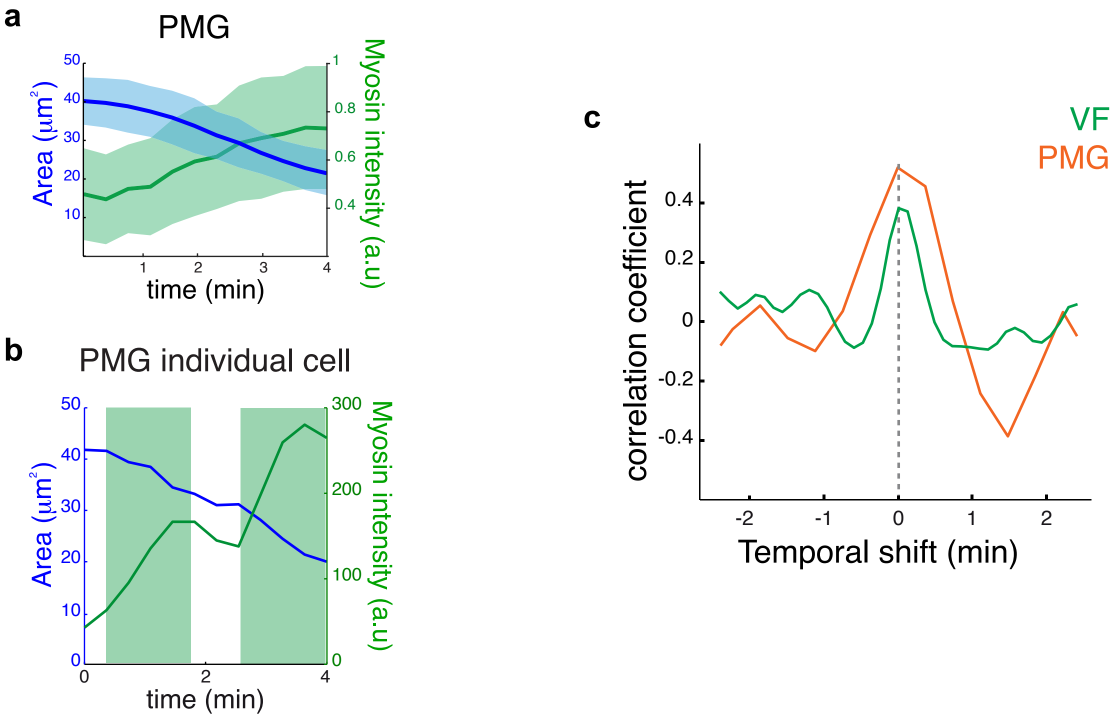

Supplementary Figure 7: **Myosin display pulsatile behavior during PMG invagination.**

a. Mean apical area (blue) and mean myosin intensity (green) over time for during PMG invagination (n=42 cells). Shaded area indicates SD.

b. Apical area (blue) and myosin intensity (green) over time in an individual representative PMG cell; contraction pulses are highlighted in green boxes.

c. Plot of averaged cross-correlation between constriction rate and myosin intensity rate of change (n = 59 VF cells, n = 42 PMG cells) against temporal shift. Note the cross-correlation peak around 0 offset for both VF (green) and PMG (orange) indicating that myosin pulses correlate with a reduction in apical area.

Supplementary Methods

Biophysical Model of Dynamic Actomyosin Arrays in the Apical Cortex

To investigate the interactions between actomyosin meshworks in the medioapical cortex and external tension anisotropy, we adapted a two-dimensional (2D) model of F-actin and myosin filaments that represent "search, capture, remodel, and traffic" processes that shape the medioapical and lateral cell cortex (Miller, C. J., Harris, D., Weaver, R., Ermentrout, B. & Davidson, L. A., manuscript in preparation). We implemented 2D numerical simulations based on an earlier "remodel and traffic" model[1]. In brief, we describe both the basic principles of the model and the extensions developed to simulate actomyosin networks within different boundaries conditions to model different resistance of the surrounding tissue.

Basic model of interacting networks of F-actin and myosin filaments.

Model elements:

Actin filaments, or filaments, are represented by 1 μm-long cylindrical rods that are polarized with a plus-, or barbed-end and minus-end. Motors move toward the filament's plus-end. To simplify F-actin polymerization, we specify a turnover rate at which a random depolymerization event occurs and new filament reappears at a random position (Table 1). Motors detach if a filament they are bound to depolymerizes.

Myosin filaments, or motors, are represented as rod-like linear springs with F-actin binding sites on either end. Unattached motors diffuse throughout the simulated 2D domain and search for F-actin filaments. Motors can bind to one or two different filaments selected randomly from those filaments within their search radius. Motors attached to filaments traffic toward the plus-end of the filament. Motors detach from a filament once the bound end of the motor reaches the plus end or may randomly detach (e.g. at a defined rate of detachment) before reaching the end of the filament. Actin filaments bound to one or more doubly-bound motors can rotate and translocate in response to forces generated by the attached motor(s). Motor

movement, force generation, and subsequent filament network remodeling is the result of ATPase activity of myosin II but we do not model this feature explicitly. Parameters used in these simulations are based on experimentally measured values and reasonable estimates for protein dynamics (see Table 1).

Model Dynamics:

We simulate the apical cortex within a single cell by considering actomyosin dynamics within a boundary domain (near the edge of the cell) and within a central domain. Throughout the cell, forces on each filament are generated by doubly-bound motors, but within the boundary domain an additional force is added to simulate tension from surrounding tissues (see following section). Before investigating complex dynamics, we first consider the generic effect of point forces on filaments. A point force from one motor can cause the attached filament to rotate and translate in a viscous medium representing the cytoplasm. For simplicity, we assume the filaments do not bend or interact with each other. Simulations begin with a random distribution of motors and filaments; filament orientations are also randomly distributed. Filaments and motor movements follow Langevin dynamics. Filament and motor biochemical interactions are implemented in a Monte Carlo framework. At each time-step, free diffusing and singly-bound motors search for filaments. Singly- and doubly-bound motor ends move along each bound filament at a fixed velocity (Supplementary Table1). Each doubly-bound motor applies a force-couple through their attachment sites to each bound filament. We calculate net translational and rotational force vectors on each filament based upon the location, orientation, and extension of all doubly-bound motors. To calculate the forces from all doubly-bound motors *j* acting on filaments *i* we first represent a single motor binding at specific position (a_j, b_j) along the length (len_i) of a filament whose center of mass is located at (x_i, y_i) with a specific orientation (θ_i) (see Supplementary Table 2 for symbol description).

(1)

$$a_j = x_i + len_j * \cos(\theta_i)$$

$$b_j = y_i + len_j * \sin(\theta_i)$$

Forces on each filament are then calculated from each attached spring-like motor *j* with a stiffness, *k*, where the other end of the bound motor is located at (A_j, B_j):

(2)

$$F_j = \begin{bmatrix} k * (A_j - a_j) \\ k * (B_j - b_j) \end{bmatrix}$$

We wish to calculate the viscous drag in the reference frame of the filament so we transform position and forces using the following rotation matrix:

(3)

$$XR_i = \begin{bmatrix} \cos(\theta_i) & \sin(\theta_i) \\ -\sin(\theta_i) & \cos(\theta_i) \end{bmatrix} \begin{bmatrix} x_i \\ y_i \end{bmatrix}$$

$$FR_j = \begin{bmatrix} \cos(\theta_i) & \sin(\theta_i) \\ -\sin(\theta_i) & \cos(\theta_i) \end{bmatrix} F_j$$

Filament velocities are calculated from the balance between parallel, perpendicular, rotational viscous drag (Γ_{perp}, Γ_{par}, and Γ_{rot}, respectively) and parallel and perpendicular forces, and rotational torque. To calculate drag, we assume the F-actin filament is a cylinder in a viscous medium, where *p* is the ratio of the filament length (*L*) to its diameter (*di*), and $\gamma_{perp} = 0.84, \gamma_{par} = 0.114$, and $\gamma_{rot} = -0.662$ are geometrical constants when $p = \infty$[2]. We further assume actin filaments experience high shear, so the dynamic viscosity, η, is higher than water.

(4)

$$\Gamma_{perp} = \frac{4\pi\eta L}{\log p + \gamma_{perp}}$$

$$\Gamma_{par} = \frac{2\pi\eta L}{\log p + \gamma_{par}}$$

$$\Gamma_{rot} = \frac{\frac{1}{3}\pi\eta L^3}{\log p + \gamma_{rot}}$$

Next, new filament positions and orientations *($XR_{ni}(1)$, $XR_{ni}(2)$, θ_{ni})* in the filament frame of reference are calculated based on the parallel *($FR_j(1)$)* and perpendicular *($FR_j(2)$)* forces, and torques *(len_j*$FR_j(2)$)* applied by the sum of attached motors.

(5)

$$XR_{n_i}(1) = XR_i(1) + dt * \frac{1}{\Gamma_{par}} \sum_j FR_j(1)$$

$$XR_{n_i}(2) = XR_i(2) + dt * \frac{1}{\Gamma_{perp}} \sum_j FR_j(2)$$

$$\theta_{n_i} = \theta_i + dt * \frac{1}{\Gamma_{rot}} \sum_j len_j * FR_j(2)$$

Finally, we transform filament positions and orientations back into the original laboratory frame of reference.

(6)

$$\begin{bmatrix} x_i \\ y_i \end{bmatrix} = \begin{bmatrix} \cos(\theta_i) & -\sin(\theta_i) \\ \sin(\theta_i) & \cos(\theta_i) \end{bmatrix} XR_{n_i}$$

$$\theta_i = \theta_{n_i}$$

Constraints on cell and tissue asymmetry imposed during ventral furrow formation:

To test the contribution of complex boundary conditions during ventral furrow formation to actomyosin meshwork dynamics we chose to embed our simulation within a polygon bounded space representing cell-cell junctions. To transmit tension between actomyosin meshworks and adjacent tissues we implemented a boundary potential that transiently connects free filament plus-ends near the boundary to the edge of the domain. In the boundary region (0.5 μm from the simulation boundary), there are boundary connections that will attach to the plus-end of filaments within the region. At each time-step, the boundary connections search for filaments within the edge domain and if found, attach a spring-like element connecting the

filament to a boundary node. All previously bound filaments are checked to determine whether they remain near the edge and detach if they are not. Filaments within this edge domain behave as other free filaments in the simulation, e.g. they are subject to depolymerization and are free to move under the action of attached motors, but experience an additional spring-like tension based on their distance from the boundary. Our implementation is not significantly different from methods used to simulate stable cross-linked actin meshworks under tension[3]. The magnitude of that tension reflects the combined compliance and stress of surrounding tissues and enables simulation of variable isotropic and anisotropic tensions.

Our model represents the boundary with 100 discrete nodes, where each node serves as a transient binding site for filaments near the edge. Each node can bind an infinite number of spring connectors to the plus end of filaments within a nearby boundary region. Filament plus ends are connected via the spring connection to the nearest boundary node and may transfer from one node to another as they move within the boundary region. Anisotropic tension from the surrounding tissues can be simulated by spatially varying the stiffness of boundary nodes. In the case of isotropic tension each node has the same Young's modulus value. To implement anisotropic tension, we set Young's modulus in the edge nodes along the top and bottom quadrants to a different value than the Young's modulus in the edge nodes along the right and left quadrants. Tensional forces on filaments near the edge are calculated based on the Young's modulus E_{node}, thickness of the cortex th, boundary perimeter $peri$, distance from the filament plus end to the boundary node Δd_i, number of nodes N_p, and number of filaments connected to each node N_{fn}.

(7)

$$F_{boundary} = \frac{E_{node} * th * peri * \Delta d_i}{N_p * N_{fn}}$$

In the event a filament moves into the central domain or depolymerizes, its coupling to the edge is broken. In the absence of other forces, filaments near the edge move toward the edge. To

simulate surrounding tissues we varied the stiffness of boundary domains to represent Young's modulus ranging from 5 kPa to 100 kPa[4]. Spring-like attachments to the boundary serve two roles: first, they allow mechanical coupling between actomyosin meshworks and surrounding tissues, and second, allow us to measure force production by those meshworks.

Filament and motor movements and biochemical interactions evolve via a finite difference scheme according to a set of biophysical parameters (Supplementary Table 1) implemented in MATLAB (Mathworks, Natick MA). Model images and time-lapse sequences representing filaments and motors were generated using custom macros written in ImageJ[5]. Complex filament-motor behaviors emerge as filament arrays rearrange through iterative actions of motors, their tension at the edge, remodeling of filament arrays, and trafficking of motors by polarized filament arrays. Simulations last 1000 time-steps (~10 seconds).

The amount of force a single motor produce depends on how stretched it is and is therefore proportional to its length. Motor alignment was calculated for motors with a length ≥ 10 pixels representing force production ≥ 0.3 nN.

Data from the model will be made available upon request.

Supplementary Table 1: **Parameters used in model**

Parameter name	Symbol	Parameter value	*In vitro* value	Reference
Simulation Duration	-	10 seconds	~1 to 2 minutes	[6]
Number of filaments	*N*	1000		
Number of motors	*M*	5000		
Boundary connections	*Np*	100		
Boundary connection stiffness	*E*	5-100 kPa		[4,7]
Persistence length	*L*	1 µm	6 nm to 10 µm	[8]
Rate of motor detachment	p_0	1 s^{-1}	Lower than p_1	
Rate of motor attachment	p_1	10 s^{-1}	Higher than p_0	

Rate of filament polymerization	p_2	0.7 s^{-1}	0.7 to 1.2 s^{-1}	[9,10]
Time step size	h	0.01 s		
Search radius of motor	r	0.3 μm	~0.3 μm length of a single myosin II	[11]
Motor velocity	v	1 $\mu m.s^{-1}$	1-3 $\mu m.s^{-1}$	[12,13]
Dynamic viscosity	η	0.1 nN ($s.\mu m^{-2}$)	1×10^{-6} nN ($s.\mu m^{-2}$)	[14]
Motor stiffness	k	3 $nN.\mu m^{-1}$	1.875 $nN.\mu m^{-1}$ (single myosin) to 1,250 $nN.\mu m^{-1}$ (skeletal muscle)	[15-18]
Rate of motor diffusion	d	$0.02.s^{-1}$		
Mean step size of motor diffusion	μ	0.001 μm		
Diameter of actin filament	di	0.008 μm	0.006 to 0.008	[19]
Domain size	-	2 μm (circle radius)	5 μm in 2D simulation	[20]

Supplementary Table 2: **Symbols used in model equations**

Symbol	Description
N	Number of filaments
M	Number of motors
(x_i, y_i)	Position of center of mass of filament *i*
θ_i	Angle of orientation of filament *i*
(a_i, b_i)	Coordinates where motor *j* is attached to filament *i*
(A_i, B_i)	Coordinates of the end of stretched motor *j*
len_j	The distance from the center of mass of filament *i* to the attachment point of motor *j*
k	Spring stiffness constant for motors
L	Length of the filament
η	Dynamic viscosity of the media
p	Ratio of length of filament to its diameter
$\gamma_{perp} = 0.84$ $\gamma_{par} = 0.114$ $\gamma_{rot} = -0.662$	Viscous drag shape coefficients when $p = \infty$
E_{node}	Young's modulus of the node
th	Thickness of the cortex
$peri$	boundary perimeter
Δd_i	distance from the filament plus end to the boundary node
N_p	Number of nodes
N_{fn}	number of filaments connected to each node

Supplementary References

1. Miller, C. J., Bard Ermentrout, G. & Davidson, L. A. Rotational model for actin filament alignment by myosin. *J. Theor. Biol.* **300,** 344–359 (2012).
2. Tirado, M. M. & la Torre, de, J. G. Translational friction coefficients of rigid, symmetric top macromolecules. Application to circular cylinders. *The Journal of Chemical Physics* **71,** 2581 (1979).
3. Kim, T., Gardel, M. L. & Munro, E. Determinants of Fluidlike Behavior and Effective Viscosity in Cross-Linked Actin Networks. *Biophys. J.* **106,** 526–534 (2014).
4. Falzone, T. T., Oakes, P. W., Sees, J., Kovar, D. R. & Gardel, M. L. Actin Assembly Factors Regulate the Gelation Kinetics and Architecture of F-actin Networks. *Biophys. J.* **104,** 1709–1719 (2013).
5. Rueden, C. & Eliceiri, K. Visualization approaches for multidimensional biological image data. *BioTechniques* **43,** S31–S36 (2007).
6. Dassow, von, M., Miller, C. J. & Davidson, L. A. Biomechanics and the Thermotolerance of Development. *PLoS ONE* **9,** e95670 (2014).
7. Alonso, J. L. & Goldmann, W. H. Feeling the forces: atomic force microscopy in cell biology. *Life Sciences* **72,** 2553–2560 (2003).
8. Murrell, M. P. & Gardel, M. L. F-actin buckling coordinates contractility and severing in a biomimetic actomyosin cortex. *Proceedings of the National Academy of Sciences* **109,** 20820–20825 (2012).
9. Amann, K. J. & Pollard, T. D. Direct real-time observation of actin filament branching mediated by Arp2/3 complex using total internal reflection fluorescence microscopy. *Proc. Natl. Acad. Sci. U.S.A.* **98,** 15009–15013 (2001).
10. Pollard, T. D. Direct measurement of actin polymerization rate constants by electron microscopy of actin filaments nucleated by isolated microvillus cores. *The Journal of Cell Biology* **88,** 654–659 (1981).
11. Shutova, M., Yang, C., Vasiliev, J. M. & Svitkina, T. Functions of Nonmuscle Myosin II in Assembly of the Cellular Contractile System. *PLoS ONE* **7,** e40814 (2012).
12. Kron, S. J. & Spudich, J. A. Fluorescent actin filaments move on myosin fixed to a glass surface. *Proc. Natl. Acad. Sci. U.S.A.* **83,** 6272–6276 (1986).
13. Murphy, C. T., Rock, R. S. & Spudich, J. A. A myosin II mutation uncouples ATPase activity from motility and shortens step size. *Nature Cell Biology* **3,** 311–315 (2001).
14. Hunt, A. J., Gittes, F. & Howard, J. The force exerted by a single kinesin molecule against a viscous load. *Biophys. J.* **67,** 766–781 (1994).
15. Neumann, T., Fauver, M. & Pollack, G. H. Elastic Properties of Isolated Thick Filaments Measured by Nanofabricated Cantilevers. *Biophys. J.* **75,** 938–947 (1998).
16. Dunaway, D., Fauver, M. & Pollack, G. Direct Measurement of Single Synthetic Vertebrate Thick Filament Elasticity Using Nanofabricated Cantilevers. *Biophys. J.* **82,** 3128–3133 (2002).
17. Nagornyak, E. M., Blyakhman, F. A. & Pollack, G. H. Stepwise Length Changes in Single Invertebrate Thick Filaments. *Biophys. J.* **89,** 3269–3276 (2005).
18. Kaya, M. & Higuchi, H. Nonlinear Elasticity and an 8-nm Working Stroke of Single Myosin Molecules in Myofilaments. *Science* **329,** 686–689 (2010).
19. Howard, J. *Mechanics of Motor Proteins and the Cytoskeleton. Sinauer Associates, Inc., Sunderland, MA.* (2001).
20. Dasanayake, N. L., Michalski, P. J. & Carlsson, A. E. General Mechanism of Actomyosin

Contractility. *Phys. Rev. Lett.* **107,** 118101 (2011).
